# HPV-related cancer prevention in Spain: progress and remaining challenges in vaccination, screening, and surveillance

**DOI:** 10.1007/s12094-026-04447-w

**Published:** 2026-06-06

**Authors:** Alejandro Gallego Martínez, Antonio Gonzalez-Martin, Luis Chiva de Agustin

**Affiliations:** 1 Department of Medical Oncology, Cancer Center Clínica Universidad de Navarra (CCUN), Calle Marquesado de Santa Marta, 1, 28027 Madrid, Spain; 2 Department of Gynecology and Obstetrics, Cancer Center Clínica Universidad de Navarra (CCUN), Madrid, Spain

**Keywords:** Human papillomavirus, Vaccination, Screening, Cervical cancer

## Abstract

Human papillomavirus (HPV) is a necessary causal factor for most cervical cancers and contributes to several other HPV-related malignancies. The nonavalent HPV vaccine targets the most prevalent oncogenic genotypes and has the potential to prevent up to 90% of HPV-attributable cancers. However, cervical cancer screening remains essential, as vaccination does not cover all oncogenic HPV genotypes, does not treat established HPV infection, and cannot prevent disease in individuals after HPV exposure. Available Spanish data do not yet show a clear sustained decline in HPV-related cancers comparable to that reported in some other high-income countries. This narrative review examines HPV vaccination, cervical cancer screening, and the burden of HPV-related cancers in Spain, with a focus on implementation gaps, surveillance limitations, and opportunities for improvement. Strengthening vaccination coverage, population-based screening, follow-up after abnormal results, and national data integration will be essential to accelerate progress toward cervical cancer elimination.

## Introduction

**Fig. 1:**
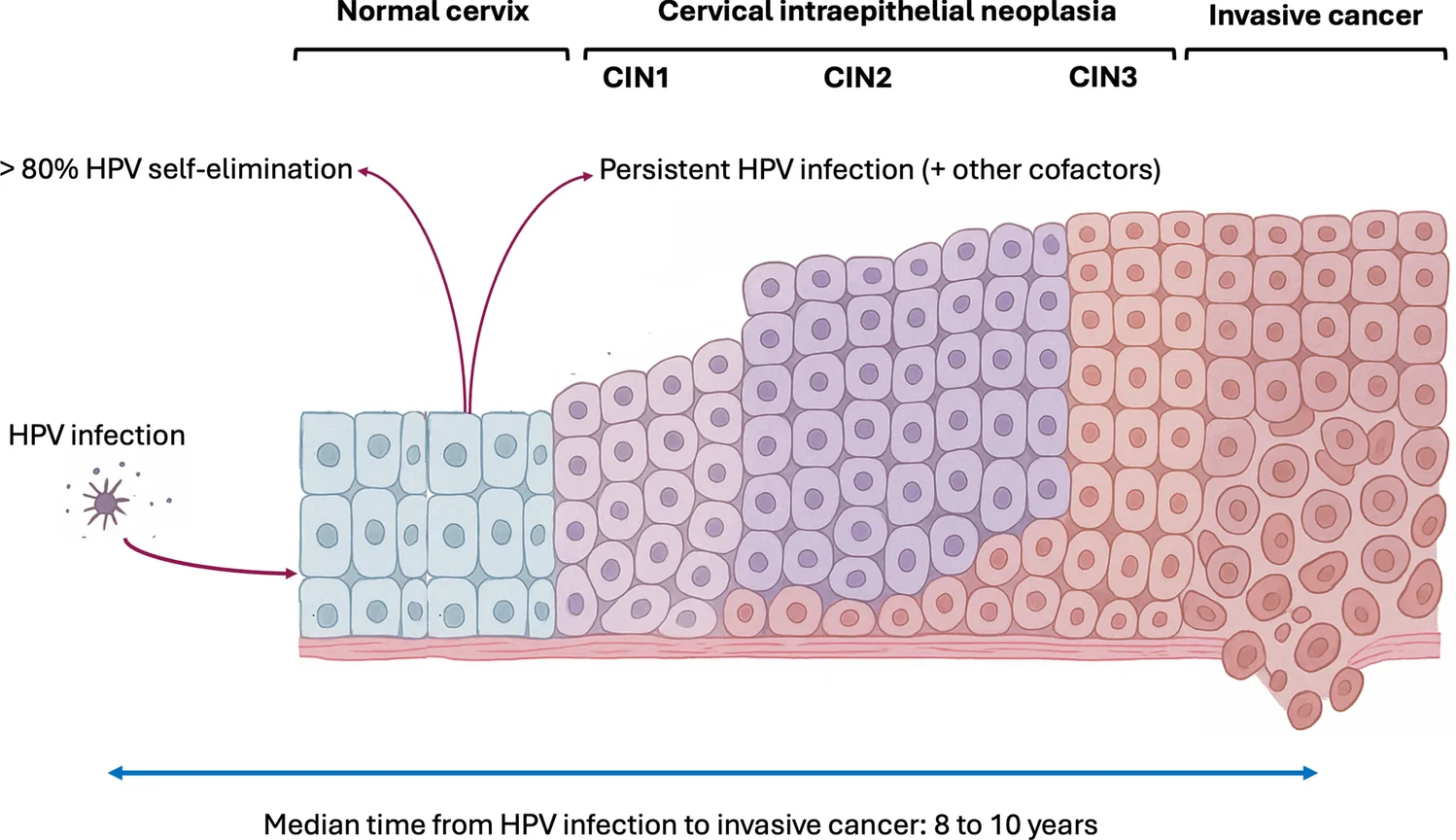
HPV carcinogenesis: from infection to CIN and invasive cancer

Human papillomavirus (HPV) infection is one of the most common sexually transmitted infections worldwide. Although most HPV infections are transient, more than 90% become undetectable within 2 years, with younger individuals having a higher probability of clearing the infection than older individuals [1, 2]. However, persistent infection with high-risk HPV genotypes may lead to precursor lesions and, over time, invasive cancer [3]. The process of HPV-related carcinogenesis is shown in Fig. 1. HPV is a necessary causal factor for the development of most cervical cancers. In addition, it increases the risk of several other malignancies, including vulvar, vaginal, anal, penile, and oropharyngeal cancers. Among the oncogenic HPV genotypes, HPV type 16 is the most prevalent and is responsible for at least 58% of cervical cancer cases in Spain [1, 4].

The prevention of HPV-related disease has been transformed by the availability of prophylactic HPV vaccines and cervical cancer screening. Current nonavalent HPV vaccine target the genotypes responsible for most HPV-attributable cancers [5, 6]. Nevertheless, vaccination does not eliminate the need for cervical cancer screening, even among vaccinated women [7, 8]. The incorporation of high-risk human papillomavirus (HR-HPV) testing has improved the sensitivity of screening [9–11]. These advances in primary and secondary prevention have led the World Health Organization to define cervical cancer elimination as a global public health goal, based on the 90–70–90 targets: 90% of girls fully vaccinated against HPV by 15 years of age, 70% of women screened with a high-performance test, and 90% of women with precancerous lesions or invasive cancer receiving appropriate treatment.

Several high-income countries have reported substantial reductions in HPV infection, high-grade cervical lesions, and cervical cancer incidence after the implementation of organized HPV vaccination programs. For example, population-based studies from England and Sweden, among others, have shown marked reductions in cervical cancer or cervical precancerous lesions [12, 13]. However, international comparisons require careful contextualization, because countries differ in vaccination timing, target populations, delivery strategies, screening organization and registry quality.

In Spain, systematic HPV vaccination for adolescent girls was implemented across autonomous communities from 2008 onward, while the nonavalent vaccine became available after its approval by the European Medicines Agency in 2015. The program was expanded to include 12-year-old boys in 2022 [14]. Cervical cancer screening is also moving toward organized population-based HR-HPV testing, although implementation remain heterogeneous across regions [15]. Despite these advances, available estimates do not yet show a clear sustained decline in HPV-related cancers in Spain [4, 16, 17].

This narrative review aims to examine HPV vaccination, cervical cancer screening, and the burden of HPV-related cancers in Spain. More specifically, it discusses why available data do not yet demonstrate a clear sustained decline in HPV-related cancers in Spain and identifies key gaps and opportunities to improve prevention strategies, surveillance systems, and progress toward cervical cancer elimination.

## Methods

This article was designed as a narrative review aimed at providing an integrated and critical overview of HPV vaccination, cervical cancer screening, and the burden of HPV-related cancers in Spain. The objective was not to conduct a formal systematic review or meta-analysis, but to summarize and interpret the available evidence in order to identify potential reasons why HPV-related cancers have not yet shown a clear sustained decline in Spain despite the availability of effective prevention strategies.

A structured literature search was conducted in PubMed to identify relevant peer-reviewed publications addressing HPV infection, HPV vaccination, cervical cancer screening, HPV-related malignancies, and epidemiological trends. Additional targeted searches were performed to identify studies reporting the population-level impact of HPV vaccination and cervical cancer screening in other high-income countries used for contextual comparison.

In addition to peer-reviewed literature, official epidemiological and public health sources were reviewed. These included reports and recommendations from the World Health Organization (WHO), the International Agency for Research on Cancer (IARC), the ICO/IARC HPV Information Centre, the Spanish Ministry of Health, the Spanish National Institute of Statistics (INE), reports from the Spanish Society of Medical Oncology (SEOM), the Spanish Network of Cancer Registries (REDECAN), the European Society of Gynecological Cancer (ESGO), and selected international public health agencies and guidelines from countries, such as the United States, Australia, and the United Kingdom [4, 14, 16–22].

Eligible sources included original studies, systematic reviews, meta-analyses, population-based cohort studies, clinical guidelines, national vaccination and screening recommendations, and official epidemiological reports addressing HPV infection, HPV vaccination, cervical cancer screening, HPV-related cancer incidence, or HPV-related cancer mortality [12, 13, 23]. The search prioritized publications and reports from the last 10 years. Older references were included when considered necessary to describe foundational aspects of HPV natural history, cervical carcinogenesis, vaccine development, or the historical evolution of cervical cancer screening [1, 3, 10].

Because this was a narrative review, no formal risk-of-bias assessment, protocol registration, PRISMA flow diagram, or quantitative synthesis was performed.

## HPV-related disease burden in Spain

HPV infection is the most common sexually transmitted infection worldwide. Low-risk genotypes, particularly HPV 6 and 11, are responsible for most genital warts, whereas persistent infection with high-risk HPV genotypes is associated with cervical cancer and several other HPV-related malignancies.

### Cervical cancer incidence and mortality in Spain

HPV is responsible for the vast majority of cervical cancer cases, and any underestimation of its attributable role is likely influenced by differences in HPV detection methods, tumor testing, and completeness of genotype information [4].

In Spain, the SEOM, based on data from the REDECAN, estimated 2307 new cervical cancer cases in 2025. By comparison, estimated cases were 2399 in 2015 and 1956 in 2020 [16].

In 2020, cervical cancer incidence in Spain was very low among women under 30 (3.33%), increased steadily from 30 to 40 (15.7%), and peaked between 40 and 49 years, where it accounted for over 26.3% of all cases. After 60, incidence gradually declined, although a notable number of cases were still observed in women over 80 (9%) [4].

### Burden of non-cervical HPV-related cancers

HPV is also associated with several non-cervical malignancies, including vulvar, vaginal, anal, penile, oral cavity, oropharyngeal, nasopharyngeal, hypopharyngeal, and laryngeal cancers.

According to 2022 estimates from the IARC and published HPV-attributable fractions by tumor type, approximately 5034 cancer cases in Spain may be considered HPV-related in the dataset used for this review, including 2927 cases in women and 2107 cases in men.

Table 1 presents the incidence of selected HPV-related cancers, the estimated proportion attributable to HPV, and their distribution by sex in Spain. Cervical cancer accounted for the largest number of HPV-related cases among women. However, other tumors also contributed meaningfully to the overall burden, including vaginal cancer (74%), anal cancer (88%), penile cancer (60%), and oropharyngeal cancer (61.4%,) [17, 24–32].

**Table 1:** Incidence of HPV-related cancers, proportion attributable to HPV, and distribution by sex in Spain

Cancer type	Total incidence (irrespective of HPV)	% HPV-association (references)	HPV-related cancers	Cases in women		Cases in men	
				Total	HPV-related	Total	HPV-related
Cervical	2020	90% [4]	1818	2020	1818	–	–
Vulvar	925	39.7% [24]	367	925	367	–	–
Vaginal	196	74% [25]	145	196	145	–	–
Penile	482	60% [26]	289	–	–	482	289
Anal	591	88% [27]	520	288	253	303	267
Oral cavity	4003	6% [28]	240	1431	86	2572	154
Oropharynx	1103	61.4% [29]	677	220	135	883	542
Nasopharynx	417	0.18% [30]	1	92	0	325	1
Hypopharynx	670	18.7% [31]	125	71	13	599	112
Larynx	3042	28% [32]	852	391	110	2651	742
HPV-related cancers			5.034		2.927		2.107

### Mortality attributable to HPV-related cancers in Spain

Based on mortality data for Spain in 2020 and estimated HPV-attributable fractions by tumor type, approximately 2000 deaths may be attributed to HPV-related cancers, nearly half were due to cervical cancer, with 1,185 occurring in women and 815 in men; [4].

Of the 372 vulvar cancer deaths, an estimated 148 were attributable to HPV. Similarly, HPV was responsible for approximately 46 of the 62 vaginal cancer deaths. Among men, penile cancer caused an estimated 71 HPV-related deaths out of 119 in total. Anal cancer, with a high HPV-attributable fraction, accounted for an estimated 122 deaths, including 66 in men and 56 in women.

For head and neck cancers, the HPV burden was also substantial. Approximately 370 oropharyngeal cancer deaths were attributable to HPV (311 in men and 60 in women). HPV was also linked to 352 of the 1141 laryngeal cancer deaths (320 in men and 33 in women). Finally, around 76 deaths from oral cavity cancer were associated with HPV [17, 24–32].

## HPV vaccination in Spain: progress and current implementation

HPV vaccination is the cornerstone of primary prevention against HPV-related disease. HPV vaccines reduce the risk of HPV infection, genital warts, cervical intraepithelial neoplasia grades 2 and 3 (CIN2/3), cervical cancer, and other HPV-related malignancies, including vulvar, vaginal, anal, penile, and oropharyngeal cancers. The nonavalent vaccine, currently the most widely used in Spain, provides the broadest genotype coverage, including HPV types 6, 11, 16, 18, 31, 33, 45, 52, and 58 [5, 6].

Vaccination is most effective when administered before HPV exposure. In HPV-naïve individuals, efficacy against vaccine-type infections ranges from 97 to 100%, supporting vaccination during early adolescence, before sexual debut [5, 6, 33]. The estimated median age at HPV acquisition is 23.9 years [34], while in Spain, the median age at first sexual intercourse has been reported to range from 17.0 to 18.0 years in men and from 16.5 to 22.7 years in women. In addition, approximately 24% of men and 19% of women aged 15 years or younger in Spain report having initiated sexual activity [4].

HPV vaccines are prophylactic and not therapeutic. They do not clear established HPV infection or treat HPV-associated dysplastic lesions. However, previous HPV infection or HPV-related disease is not a contraindication to vaccination, since vaccination may still protect against HPV genotypes to which the individual has not yet been exposed. This is clinically relevant, because co-infection with multiple HPV genotypes is common; in one study of 4244 HPV-positive women tested for 14 genotypes, co-infection was found in 22.5% of participants [35].

Adjuvant HPV vaccination after surgical treatment of CIN2/3 may reduce the risk of recurrent or persistent high-grade lesions. A meta-analysis of 11 studies including 21, 310 patients reported a 65% reduction in recurrent or persistent CIN2/3 among vaccinated patients compared with surgery alone, and another systematic review of 22 studies supported this benefit, particularly for lesions associated with HPV 16 and 18 [7]. However, there is currently no evidence supporting prophylactic HPV vaccination as a therapeutic intervention in patients already diagnosed with HPV-related gynecologic cancers, and the European Society of Gynecological Oncology (ESGO) prevention committee can not recommend its use in this setting because of insufficient evidence [8].

The safety profile of HPV vaccines supports their use as a population-level prevention strategy. Large clinical trials, systematic reviews, and post-marketing surveillance studies have not shown an increased risk of serious adverse events compared with unvaccinated populations or placebo groups. Mild local reactions are more frequent after vaccination, but serious safety signals have not been consistently identified [6, 36, 37].

### Evolution of HPV vaccination recommendations in Spain

In Spain, systematic HPV vaccination for adolescent girls was approved in November 2007 and implemented across regions from 2008 onward. Initially bivalent and quadrivalent vaccines were used. The nonavalent vaccine became available later, after its approval by the European Medicines Agency in 2015.

The program was expanded in October 2022 to include 12-year-old boys, representing an important step toward gender-neutral HPV prevention. More recently, in 2024, the Spanish Ministry of Health updated its recommendations, incorporating a single-dose schedule for girls and boys at 12 years of age and for unvaccinated individuals up to 18 years [14].

This change is supported by emerging evidence on reduced-dose schedules. In the large randomized noninferiority ESCUDDO trial, which included 20,330 girls aged 12–16 years, a single dose of either the bivalent or nonavalent HPV vaccine was found to be noninferior to two doses for preventing persistent HPV16 or HPV18 infection [5]. Nevertheless, multi-dose schedules remain recommended for selected higher-risk populations, including immunosuppressed individuals and women undergoing treatment for CIN2/3 [14]. In routine clinical practice, the nonavalent vaccine is currently the most widely used HPV vaccine in high-income countries, including Spain. For individuals outside publicly funded indications, vaccination must be assumed privately, which may represent a potential access barrier in some groups. In Spain, the cost per dose is 172.55€ for Gardasil 9®.

### Vaccination coverage and regional heterogeneity

**Fig. 2:**
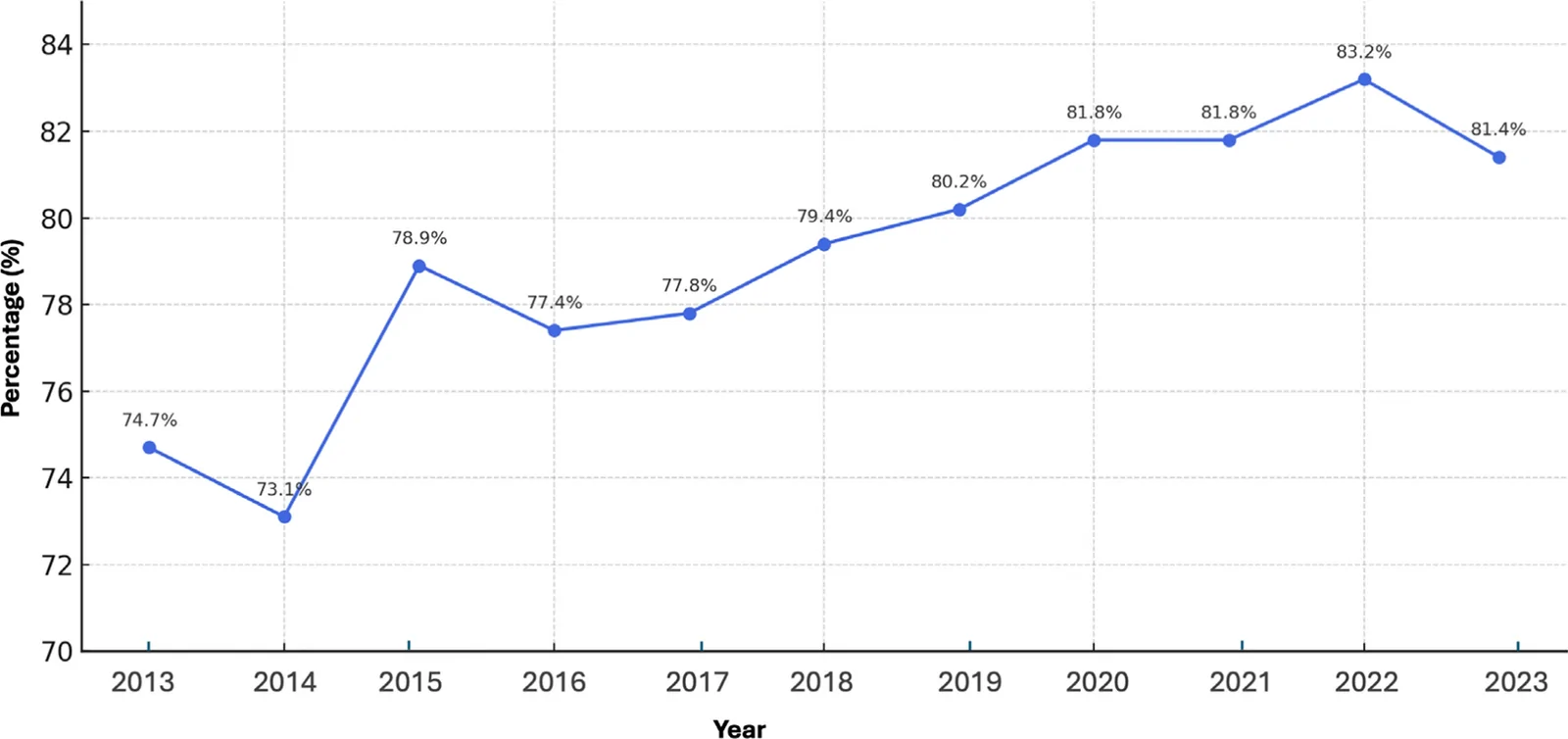
HPV vaccination coverage among women in Spain from 2013 to 2023, according to data from the Spanish National Institute of Statistics (INE)

According to national data summarized in this review, HPV vaccination coverage among adolescent girls in Spain fluctuated between 74.7% in 2013 and 81.4% in 2023 [20]. Although these figures indicate substantial vaccine uptake, they remain below the WHO target. Figure 2 illustrates HPV vaccination coverage among adolescent girls in Spain from 2013 to 2023.

There is considerable variability in coverage across regions, likely influenced by differences in data collection and reporting systems. For example, in Ceuta, vaccination coverage has been reported as low as 11.4% to 23.2% in recent years, whereas coverage remained consistently above 80% from 2013 to 2019. In addition, data from the Balearic Islands show substantial gaps, with missing information for six of the past 10 years reported.

In contrast, regions such as Navarra and the Basque Country have demonstrated consistently high coverage in recent years. Canary Islands have reported persistently low coverage, falling below 70% since 2020 [20].

### Vaccine genotype coverage and residual risk

**Fig. 3:**
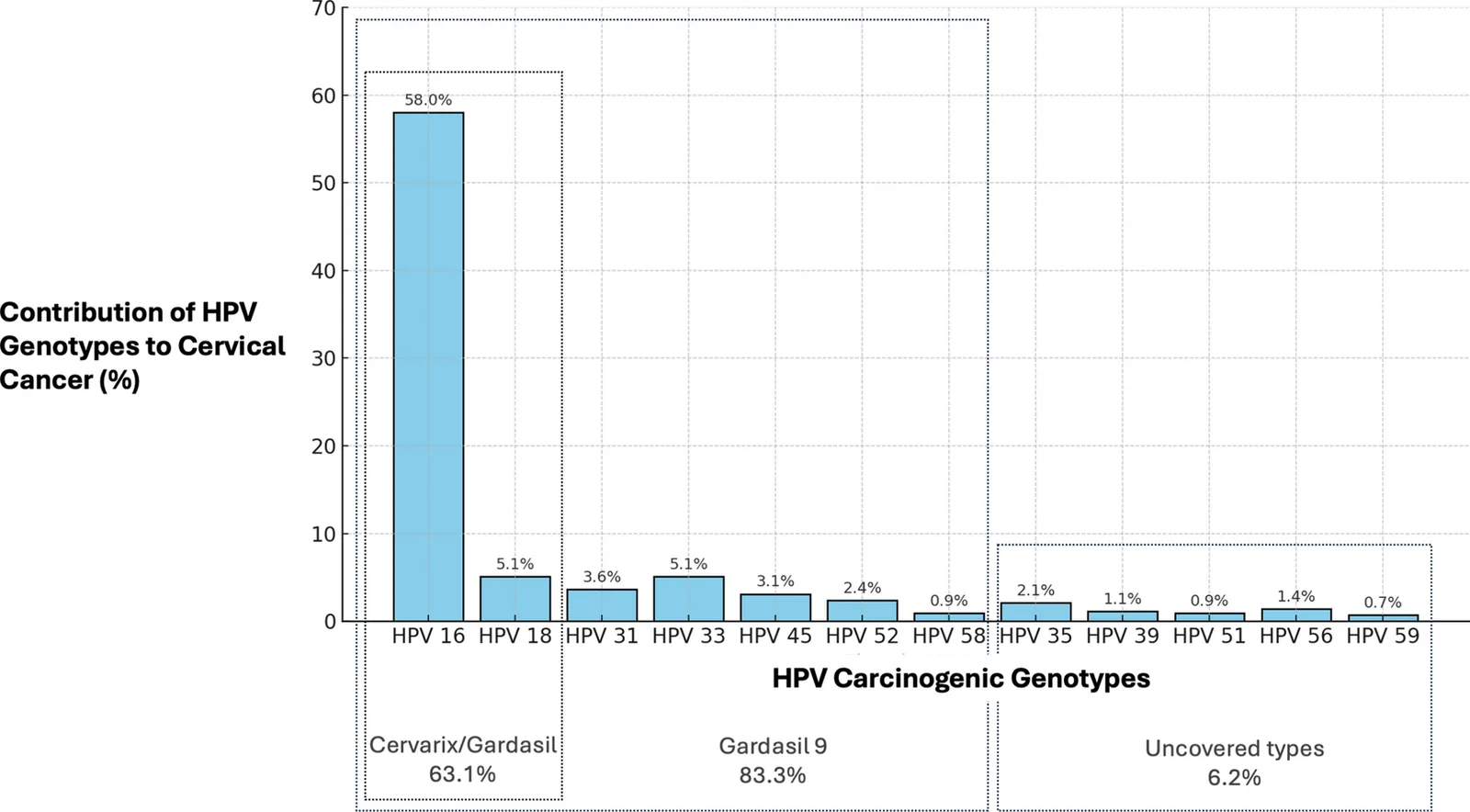
Distribution of carcinogenic HPV genotypes (possible/probable carcinogenic not included) in cervical cancer cases in Spain and their coverage by HPV approved vaccines

The potential impact of HPV vaccination in Spain is supported by the distribution of HPV genotypes in cervical cancer. As shown in Fig. 3, most cervical cancer cases in Spain with available genotype information are associated with HPV types covered by currently approved vaccines, particularly the nonavalent vaccine [4].

HPV genotype information is available for only 89.5% of cervical cancer cases in Spain. Among cases with available data, approximately 83.3% are attributed to HPV genotypes covered by the nonavalent vaccine, while 6.2% are associated with non-vaccine types, leaving 10.5% of cases without subtype-specific HPV information or confirmed HPV attribution. This gap is most likely attributable to missing diagnostic [4].

## Cervical cancer screening in Spain: recommendations, implementation, and gaps

Cervical cancer screening remains the main secondary prevention strategy for HPV-related disease. Although HPV vaccination substantially reduces the risk of infection with vaccine-covered genotypes, screening continues to be essential. Current vaccines do not cover all oncogenic HPV types, many women currently at risk of cervical cancer were not vaccinated during adolescence or were vaccinated after possible HPV exposure [7, 8].

HPV infection is a necessary but not sufficient cause of cervical cancer. Several factors influence the risk of persistent HPV infection and progression to CIN and invasive cervical cancer, including immunosuppression, particularly HIV infection, smoking, and HPV genotype, especially HPV 16 and 18.

The development of cervical cancer is usually slow and progressive, as illustrated in Fig. 1. HPV infection initially targets basal epithelial cells of the cervix, most commonly at the ectocervical–endocervical junction. Progression from initial HPV infection to microscopic evidence of premalignant lesions may take approximately 5 years, while progression to invasive cervical cancer may occur over a period of up to 10 years. High-grade cervical lesions may progress to invasive cancer within 3–7 years if left untreated [3].

### Screening modalities and the role of HR-HPV testing

Cervical cancer screening has progressively evolved from cytology-based strategies toward high-risk human papillomavirus (HR-HPV) testing. Cytology has historically been the basis of cervical cancer screening, but its sensitivity for detecting high-grade cervical lesions is lower than that of HR-HPV testing [25, 26]. HR-HPV testing provides higher sensitivity for the detection of CIN2/3 Reported sensitivity values are between 93 and 97% for detecting CIN2/3 lesions, respectively (27) [26, 27].However, in women under 30 years of age, cytology is generally preferred because of the higher prevalence of transient HPV infection, which may lead to false-positive findings with HPV testing [9–11, 15].

Most screening HPV tests detect at least the 12 high-risk oncogenic genotypes. False-negative results may still occur for several reasons, like the presence of HPV types not detectable by current testing methods or issues related to sample collection or laboratory processing, among others.

However, HR-HPV testing also requires appropriate triage. If an HPV test has a positive result, does not necessarily indicate clinically relevant disease a triage test (typically cytology) is recommended to stratify risk and identify only those women most likely to have clinically relevant lesions. However, in the presence of HPV types 16 or 18, patient is referred directly to colposcopy [15].

Self-sampling for HR-HPV testing may also represent an opportunity to improve participation among under-screened women. This approach may be particularly useful for women who face barriers to gynecological examination, have limited access to healthcare, or are less likely to participate in conventional screening programs.

### Current cervical cancer screening recommendations in Spain

Spain has progressively incorporated high-risk human papillomavirus (HR-HPV) testing into cervical cancer screening and is currently transitioning from opportunistic screening toward an organized population-based program. According to the 2024 consensus document for the implementation of population-based cervical cancer screening in the Spanish National Health System, screening recommendations are stratified by age and HPV vaccination status.

For women aged 25–29 years without adequate vaccination protection, cytology every 3 years is recommended. Women vaccinated against HPV before the age of 15 may start screening at 30 years of age. From 30 to 65 years of age, HR-HPV testing every 5 years is recommended, regardless of vaccination status. If the HR-HPV test is positive, appropriate triage should be performed to stratify the risk of clinically relevant cervical lesions and determine whether colposcopy, repeat testing, or surveillance is required [15].

A key objective of the current Spanish strategy is to implement these recommendations within an organized population-based screening framework. Implementation, however, remains heterogeneous. Spain’s National Health System is decentralized, and regions are responsible for operationalizing national recommendations.

### Screening coverage in Spain: uncertain national estimates

Assessing cervical cancer screening coverage in Spain remains challenging. According to data from the ICO/IARC HPV Information Centre, among women aged 25–65 years, approximately 87% were estimated to have ever been screened, 80% to have been screened in the previous 5 years, and 72% in the previous 3 years [4]. However, these figures should be interpreted cautiously, because the source, completeness, and comparability of the data are uncertain.

Several factors limit the interpretation of national screening estimates. Cervical cancer screening in Spain is coordinated by the Ministry of Health in collaboration with the 17 autonomous communities and 2 autonomous cities, which may lead to variability in program implementation, data collection, and reporting. In addition, screening may be performed in both public and private healthcare settings, and private-sector screening may not be fully integrated into public health information systems. Therefore, the exact coverage of cervical cancer screening in Spain remains uncertain.

Regional data suggest that lack of previous screening remains an important problem among women who develop cervical cancer. In a retrospective study conducted in Asturias among 374 women diagnosed with cervical cancer between 2000 and 2010, 60.7% had no documented history of prior cervical cancer screening [38].

National data on follow-up after abnormal screening results are also limited. This is a major gap, because incomplete follow-up may disproportionately affect women with social vulnerability, migrant populations, women without stable access to healthcare, and those screened outside organized public programs.

## International context: HPV prevention outcomes in other high-income countries

### Impact of HPV vaccination on infection, genital warts, and cervical precancer

Several high-income countries have reported measurable reductions in HPV-related outcomes after the implementation of organized HPV vaccination programs. These reductions have generally appeared first in intermediate endpoints, such as vaccine-type HPV infection, genital warts, and high-grade cervical intraepithelial neoplasia, before becoming evident in invasive cervical cancer incidence. Reduction in HPV infection provide early evidence of vaccine impact [12, 13, 33].

Genital warts represent another early marker of vaccine impact, particularly for vaccines that include HPV types 6 and 11. Although the 2023 Report of Sexually Transmitted Infections in Spain does not specify the incidence of genital warts, international experience is informative. In countries such as the United Kingdom, genital warts have historically represented one of the most commonly diagnosed sexually transmitted infections, and reductions after HPV vaccination have been used as an early indicator of population-level vaccine effectiveness [39].

### Evidence of declining cervical cancer incidence

More recently, several high-income countries have reported reductions in invasive cervical cancer incidence among vaccinated cohorts. Two landmark population-based studies from Sweden and England illustrate the population-level impact of HPV vaccination, particularly when administered before likely HPV exposure.

In Sweden, quadrivalent HPV vaccination was associated with a significantly lower risk of invasive cervical cancer before the age of 31 years. The cumulative incidence was lower among vaccinated than unvaccinated women, and the adjusted risk of cervical cancer was reduced by 63% in vaccinated individuals. The effect was strongest among women vaccinated before 17 years of age, in whom the estimated risk reduction reached 88%, compared with 53% among women vaccinated between 17 and 30 years of age [13].

Similarly, in England, a population-based study including 13.7 million person-years of follow-up among women aged 20 to younger than 30 years showed a marked reduction in cervical cancer and CIN3 incidence after the introduction of the national HPV vaccination program. The relative reduction in cervical cancer incidence was 34% when vaccination was offered at 16–18 years, 62% when offered at 14–16 years, and 87% when offered at 12–13 years. The corresponding reductions in CIN3 were 39%, 75%, and 97%, respectively. By June 2019, the program was estimated to have prevented 448 cervical cancer cases and more than 17,000 CIN3 cases [12].

### International differences in HPV vaccination recommendations

HPV vaccination recommendations vary across countries, particularly regarding catch-up vaccination and vaccination in adults. In the United States, routine HPV vaccination is recommended for all individuals through age 26 years if not previously or adequately vaccinated. For adults aged 27–45 years, vaccination is not routinely recommended, but shared clinical decision-making is encouraged for individuals who may benefit from vaccination [18].

In Australia, HPV vaccination is publicly funded for individuals aged 12–25 years, while vaccination at older ages may be considered on an individual basis depending on risk factors [19].

### International differences in cervical cancer screening

Cervical cancer screening recommendations also vary across countries. In the United States, screening begins at age 21 years, regardless of the age of sexual initiation. Women aged 21–29 years are recommended to undergo cytology every 3 years. Between 30 and 65 years of age, several evidence-based options are available, including co-testing with cytology and HPV testing every 5 years, cytology alone every 3 years, or HPV testing alone every 5 years [21].

In Australia, the National Cervical Screening Program recommends initiating cervical screening at age 25 years, with primary HPV testing every 5 years, and continuing until age 74 years [22]. In Spain, current recommendations are moving toward organized population-based screening based on HR-HPV testing, with cytology still used in younger women depending on vaccination status [15].

## Discussion: why are HPV-related cancers not clearly declining in Spain?

The central question addressed in this review is why HPV-related cancers, particularly cervical cancer, have not yet shown a clear sustained decline in Spain despite the availability of effective preventive strategies. The current situation likely reflects the interplay of multiple factors, which are discussed below.

### Vaccination coverage remains below elimination targets

In Spain, HPV vaccination coverage among adolescent girls has been relatively high but remains below the WHO target of 90% vaccination by 15 years of age. National coverage fluctuated between approximately 75% and 81% from 2013 to 2023, indicating substantial uptake but also leaving a relevant proportion of adolescents unvaccinated [20].

Several factors may contribute to incomplete vaccination. Besides access and organizational barriers, vaccine hesitancy may play a role. Some individuals or families may decline HPV vaccination, because they underestimate the risk of HPV-related disease or are influenced by misinformation. Although HPV vaccines have shown a favorable safety profile in clinical trials, systematic reviews, and post-marketing surveillance studies, fear of adverse events may still affect vaccine confidence [6, 36, 37]. Healthcare professionals are essential in this context. In Spain, professional advice has been identified as an important determinant of HPV vaccination uptake. A strong recommendation from physicians, nurses, and primary care teams can improve vaccine acceptance, especially when families have doubts about safety, timing, or the need for vaccination [40].

Migration may also influence population-level impact. Spain has a substantial immigrant population, including individuals who may have grown up in countries, where HPV vaccination was unavailable, introduced later, or had lower coverage than in Spain. As a result, some adolescents and young adults living in Spain may be unvaccinated, under-vaccinated, or have incomplete vaccination records. This is particularly relevant for catch-up strategies and equitable access to prevention.

### Screening effectiveness depends on the full pathway

Cervical cancer screening remains essential, because vaccination does not protect against all oncogenic HPV genotypes, does not treat established infection, and does not cover women who were infected before vaccination or were never vaccinated [7, 8]. However, screening effectiveness depends on completion of the entire pathway: invitation, participation, test quality, triage, colposcopy referral, treatment of CIN2/3, and post-treatment surveillance.

Several factors may limit the real-world impact of screening in Spain. Organized population-based screening has not been uniformly implemented across all regions, and private-sector screening data may not be fully integrated into public health registries. Regional heterogeneity in implementation and reporting also makes it difficult to identify gaps and compare program performance.

Thus, the challenge is not the absence of effective screening recommendations, but ensuring that the entire pathway is implemented, monitored, and completed in a standardized and equitable manner across the eligible population.

### Why vaccination and screening may not yet translate into a clear decline

Several factors may explain why HPV prevention has not yet produced a clearly measurable decline in HPV-related cancer incidence in Spain. Vaccination coverage remains below the WHO target, regional heterogeneity and some women currently developing cervical cancer were not vaccinated during adolescence [14, 20]. The delayed introduction of male in 2022 is another relevant factor [14]. The effect of male vaccination on HPV transmission, herd protection, and male HPV-related cancers cannot yet be expected to be measurable. Direct protection against anal, penile, and oropharyngeal cancers will require long-term follow-up, particularly, because these malignancies lack established population-based screening programs.

Prevention strategies must also address equity more broadly. Under-vaccination and under-screening may concentrate among populations with lower healthcare access, socioeconomic vulnerability, migration-related barriers, or limited engagement with preventive services. Improving average national coverage is important, but reducing inequalities may be equally critical to achieving a measurable reduction in cervical cancer incidence.

The natural history of cervical carcinogenesis must also be considered. Persistent HR-HPV infection may take years to progress to high-grade cervical intraepithelial neoplasia and even longer to invasive cancer [3]. Consequently, reductions in HPV infection or precancerous lesions may precede measurable reductions in invasive cancer incidence by many years.

Initiatives such as the REVIVE study (NCT07113379) may help characterize current gaps in cervical cancer prevention in Spain, particularly among women already diagnosed with cervical cancer, and may provide evidence to identify missed opportunities in vaccination, screening, follow-up, and access to care.

### Limitations of Spanish epidemiological data and surveillance gaps

A major challenge in evaluating HPV-related cancer burden in Spain is the heterogeneity and incompleteness of available data. The epidemiological picture is constructed from regional registries, international databases, vaccination coverage reports, screening recommendations, and institutional public health reports [4, 15–17, 20]. These sources are valuable but differ in methodology, timeframe, population coverage, and regional completeness.

This is particularly relevant, because the Spanish National Health System is decentralized, and implementation and data collection depend largely on the regions [14, 15, 20]. Screening data are especially difficult to interpret, as screening may occur in both public and private healthcare settings, and private-sector data may not be fully captured [4].

This discrepancy highlights the need for better indicators, not only of screening participation, but also of adherence to recommended intervals, triage after positive tests, colposcopy referral, treatment of high-grade lesions, and post-treatment surveillance.

### International comparisons should be contextualized

International experience shows that HPV vaccination can reduce HPV infection, genital warts, cervical precancer, and invasive cervical cancer when implemented with high coverage and sufficient follow-up. However, these results cannot be directly extrapolated to Spain without considering differences in vaccination timing, target age groups, catch-up policies, delivery strategies, coverage, gender-neutral vaccination, screening organization, registry completeness, and duration of follow-up [12–15, 18–21].

Therefore, the absence of a clear sustained decline in Spain should not be interpreted as evidence that Spain is performing worse than other countries or that HPV prevention strategies are ineffective. Rather, Spain may still be in a transitional phase, in which prevention strategies have been implemented but their impact is delayed, unevenly distributed, or difficult to quantify with available data.

### Limitations of this review

This review is limited by the heterogeneity of the available evidence. Spanish data on HPV vaccination coverage, cervical cancer screening participation, follow-up after abnormal results, and HPV-related cancer burden are derived from multiple sources that differ in methodology, completeness, and timeframe [4, 15, 17, 20]. Several indicators needed to assess the full prevention pathway are unavailable or not consistently reported at the national level.

Because this was a narrative review, no formal risk-of-bias assessment or quantitative synthesis was performed. However, the objective was not to provide pooled estimates, but to integrate the available evidence and identify potential explanations for the apparent gap between effective prevention strategies and current population-level trends in Spain.

For these reasons, conclusions should be interpreted cautiously. Available evidence does not allow a definitive assessment of the full population-level impact of HPV vaccination and cervical cancer screening in Spain. However, it identifies relevant gaps in implementation, monitoring, equity, and surveillance that may explain why a clear sustained decline in HPV-related cancers has not yet been observed.

## Conclusions

HPV vaccination and cervical cancer screening are highly effective strategies for preventing HPV-related disease, and Spain has progressively incorporated both approaches into national public health recommendations. However, available estimates do not yet demonstrate a clear sustained decline in HPV-related cancers comparable to that reported in some other high-income countries. This observation should be interpreted cautiously and should not be understood as evidence that vaccination or screening are ineffective.

The current situation is likely explained by the interaction of several factors: vaccination coverage below the WHO elimination target, delayed implementation of gender-neutral vaccination, heterogeneous screening implementation, incomplete follow-up indicators, fragmented data collection, and the long latency between HPV infection and invasive cancer.

To accelerate progress toward cervical cancer elimination and broader HPV-related cancer prevention, Spain should prioritize standardized national monitoring, improved integration of vaccination and screening data, consolidation of gender-neutral vaccination, reduction of regional inequalities, and specific strategies to reach under-vaccinated and under-screened populations. Strengthening these areas will be essential to ensure that the proven efficacy of HPV prevention strategies translates into measurable population-level reductions in HPV-related cancer.
